# Differences in mortality risk by levels of physical activity among persons with disabilities in South Korea

**DOI:** 10.1515/med-2024-1118

**Published:** 2024-12-31

**Authors:** SeungCheor Lee, Yeowool Lee, Saengryeol Park, So-Youn Park, In-Hwan Oh

**Affiliations:** Department of Preventive Medicine, School of Medicine, Kyung Hee University, Seoul, 02447, South Korea; Department of Health Care Administration, Seoyeong University, Gyeonggi-do, 10843, South Korea; Department of Physical Education, College of Education, Chonnam National University, Gwangju, 61186, South Korea; Department of Medical Education and Humanities, School of Medicine, Kyung Hee University, Seoul, 02447, South Korea

**Keywords:** physical activity, mortality, survival analysis, persons with disabilities, Republic of Korea

## Abstract

**Aim:**

The World Health Organization’s recommendation of at least 150 min of physical activity per week is important for increasing the lifespan of persons with disabilities (PWDs).

**Methods:**

Conduct a survival analysis to examine the relationship between physical activity and mortality using cohort data from the National Health Insurance Service in South Korea from 2017 to 2021. The survival analysis included 259,146 PWDs, with a maximum follow-up of 57 months, and adjustments for covariates, including physical activity level, comorbidities, smoking, and alcohol consumption.

**Results:**

People who exercised >150 min weekly had a lower risk of death compared to those who exercised less (adjusted hazard ratio [AHR]: 0.727, 95% confidence interval [CI]: 0.674–0.784). The risk of death increased with increasing age (AHR: 1.08, 95% CI: 1.077–1.083), smokers had a higher risk of death than non-smokers (AHR: 1.487, 95% CI: 1.396–1.583), and the risk of death increased with increasing Charlson comorbidity index scores (AHR: 1.228, 95% CI: 1.22–1.237).

**Conclusion:**

Even after adjusting for socioeconomic and other risk factors, PWDs who are physically inactive have a higher risk of death. Customized physical activity policies for PWDs are needed to reduce health inequities.

## 1 Introduction

The World Health Organization (WHO) recommends at least 150 min of physical activity per week [1]. However, according to a study that evaluated the participation of persons with disabilities (PWDs) in physical activity, 60% did not regularly meet this goal [2]. Another study reported that PWDs, compared with those without disabilities, were less likely to perform physical activity and had a higher risk of obesity, and only 39.4% of PWDs in South Korea had a body mass index within the normal range [3]. Consequently, PWDs are more likely to be at higher risk of cardiovascular disease, which can lead to premature death [4]. We already know that physical activity is beneficial to health, but there is a disparity between persons without disabilities and PWDs, and this study aims to increase physical activity among PWDs.

The most noticeable change in the recently revised WHO Physical Activity Guidelines is the warning that people of all ages, including children and adolescents, should not be sedentary [5]. In previous guidelines, the WHO suggested a minimum activity duration of 10 min for a single session, but the revised guidelines emphasize steady movement with no limit to physical activity. For your health, achieving the minimum physical activity recommendations in the guideline is most important. PWDs and prolonged sedentary behaviors, including those at high risk, pregnant women, and people with chronic diseases, should aim to achieve the same minimum of 150 min of physical activity per week as people without disabilities, and strength training is recommended at least twice a week for each body part [5]. The new revised guidelines recognize that every movement counts, and staying active is important, including taking the stairs or doing chores, to maintain health. Scientific research on the adverse health consequences of prolonged sitting is also cited [6].

Health inequality of individuals with disabilities is conceptualized as “a health gap between groups or within a group of persons with disabilities that arises as a result of unequal access to health and medical services as a result of deprivation of the right to health of persons with disabilities and continuous social discrimination against persons with disabilities, and the absence of a system to prevent and manage secondary disabilities” [7]. Therefore, PWDs are relatively vulnerable and likely to develop chronic diseases at an early age [8]. Previous studies have suggested that PWDs have a higher prevalence of chronic diseases and are more likely to have serious complications or reduced life expectancy [9]. Aside from comorbid diseases, the other main factors affecting mortality rates are health risk factors such as smoking, drinking, and physical inactivity [10]. A lack of physical activity is associated with premature death and can increase mortality in the general population [10]. A recent study reported that sufficient physical activity is related to even lower coronavirus disease-related infections and mortality [11]. However, the relationship between physical activity and mortality among PWDs has not been extensively studied.

Socioeconomic factors such as occupation, education level, and income levels are closely related to mortality [12]. In particular, minority groups may have a lower socioeconomic status than other groups because of social discrimination. In addition, the socioeconomic gap between individuals with and without disabilities may be further widened owing to limitations caused by physical or mental impairments resulting from interactions with the social environment [12]. When researching the health of PWDs, studies analyzing factors related to physical activity and death should consider both health and socioeconomic factors. Therefore, understanding and developing associations between health and socioeconomic risk factors when designing health promotion programs for PWDs may be effective in reducing mortality risk.

Until now, no studies have analyzed the relationship between health risk factors, socioeconomic factors, and mortality and focused on the amount of physical activity among PWDs in Korea. Therefore, this study investigated the association between physical activity and mortality using data from the National Health Insurance Service (NHIS), which is representative of the Korean population.

## 2 Methods

### 2.1 Data source and participants

**Figure 1:**
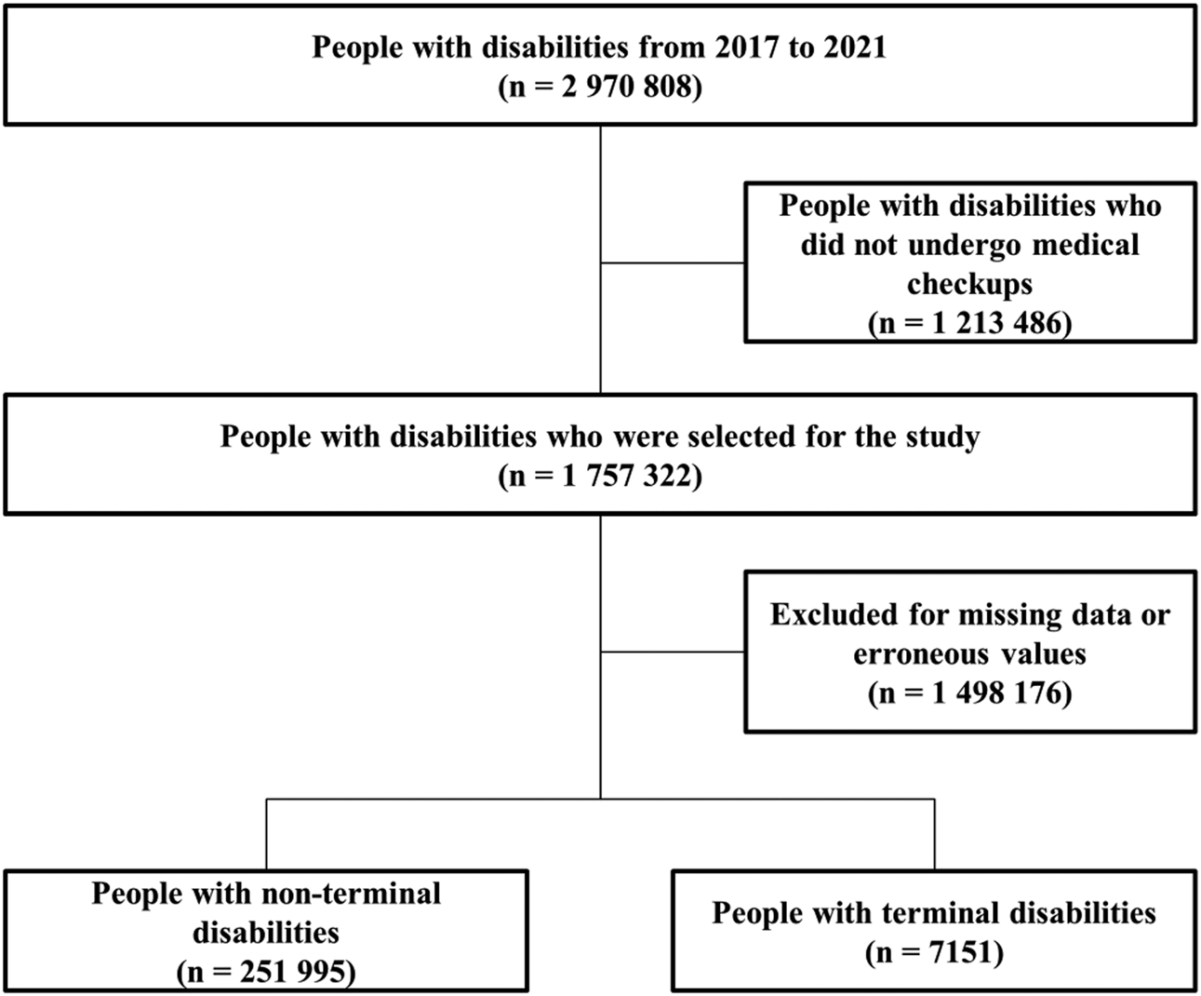
Flowchart of the selection process for analysis.

This research is an analytical study using cohort data and examined factors affecting mortality risk among PWDs using data from the NHIS, which is representative of South Korea. We used health examination records from 2017 to 2021, with a maximum follow-up period of 57 months for deaths in survival analysis. The NHIS covers approximately 97% of the South Korean population and provides mandatory health checkups every 2 years [13]. In addition, the health insurance data include information on medical treatments and health checkups, which encompasses health behaviors such as smoking, drinking, and physical activity. For this study, of 2,970,808 people with registered disabilities as of September 30, 2021, we selected 1,757,322 people who participated in health checkups provided by the NHIS. Of these, 1,498,176 were excluded because of missing data on the amount of physical activity, region of residence, income quintile, and smoking and drinking status. Therefore, 251,995 people with non-terminal disabilities and 7,151 people with terminal disabilities were selected as final participants (Figure 1). The NHIS data record patient demographics, consultation history, diagnosis history, prescriptions, and more of people who receive health screenings in the country. The dataset analyzed for this study was provided by the NHIS public database (NHIS-2022-1-223). The dataset is fee-based and was provided to the authors after a reasonable application process and approval by the NHIS. Additional access to the dataset is available with permission from the NHIS.

### 2.2 Definition of study variables

#### 2.2.1 PWDs

In South Korea, PWDs are registered with the Ministry of Health and Welfare and diagnosed by a doctor under the Disability Welfare Act, to measure the degree and type of disability. Disabilities were classified based on the severity as mild or severe; the disability type was classified as a legally prescribed physical disability, disability due to brain lesions, hearing disability, visual disability, speech disability, intellectual disability, autistic disorder, mental disorder, cardiac dysfunction, kidney dysfunction, respiratory dysfunction, hepatic dysfunction (or liver dysfunction), facial disfigurement, intestinal fistula/urinary fistula, or epilepsy [14].

#### 2.2.2 Physical activity

Physical activity levels were categorized into two groups based on WHO guidelines as follows: the physical inactivity group (i.e., less than 150 min of physical activity per week) and the physical activity attainment group (i.e., more than 150 min of physical activity per week). Physical activity was measured using a self-report questionnaire based on the NHIS health checkup information. Moderate-intensity physical activity was measured using the following question: “During the last week, how many times a week and for how many hours a day did you engage in physical activity at a moderate level for more than 10 min (e.g., fast walking, doubles tennis, riding bicycle, and cleaning)?” High-intensity physical activity was assessed using the following question: “During the last week, how many days a week and for how many hours a day did you engage in physical activity at a vigorous level for more than 10 min (e.g., running, aerobic, fast riding a bicycle)?” Physical activity level was classified as either more or less than 150 min per week [1]. Physical activity was defined based on the physical activity guidelines of at least 150 min of moderate-intensity or the equivalent high-intensity physical activity per week, where 1 min of high-intensity physical activity is equivalent to 2 min of moderate-intensity physical activity. This classification has been widely used in the literature [15].

#### 2.2.3 Covariates

We adjusted for covariates associated with mortality, including physical activity, sex, age, health insurance type, income quintile, reemployment status, smoking status, drinking status, comorbidities, and region of residence. Health insurance types were categorized as medical aid, local residents, and employed. Income levels were categorized as medical aid, 1, 2, 3, 4, and 5, with higher numbers indicating a higher income. Medical aid was provided as part of basic living assistance, and people with medical aid did not pay insurance premiums.

Reemployment referred to being reemployed as a worker in the same region. Residential areas were categorized by the city and province in South Korea. The cities were Seoul, Busan, Incheon, Gwangju, Daegu, Daejeon, Ulsan, and Sejong, and the provinces were Gangwon, Gyeonggi, Chungcheongnam, Chungcheongbuk, Gyeongsangnam, Gyeongsangbuk, Jeollanam, Jeollabuk, and Jeju.

Comorbidities were comprehensively evaluated using the Charlson comorbidity index (CCI), which can be applied to various diseases. The CCI uses administrative data as a data source and is widely used in research because it reflects the severity of comorbidities. Comorbidities refer to other diseases the patient has, aside from the main diagnosis (e.g., diabetes and hypertension). CCI scores were classified using the average scores based on the medical records.

Smoking and alcohol drinking statuses were assessed using a self-report questionnaire. Smoking status was measured using the question, “Have you ever smoked over five packs of cigarettes (100 pieces) in your lifetime?” Drinking status was assessed using the question, “How many times do you drink alcohol per week?”

### 2.3 Statistical analyses

The chi-squared test and survival analyses were used to analyze the data. Statistical analyses were performed using SAS version 9.4 (SAS Institute Inc., Cary, NC, USA), and the significance level for all statistical tests was set at 5%. The chi-squared test was performed to assess the characteristics of PWDs related to physical activity. Survival analysis was performed to analyze the factors affecting the mortality risk of PWDs. The Cox proportional hazard model was used by setting survival and death as dependent variables. The study’s maximum follow-up period was 57 months.


**Informed consent:** Since de-identified data were used, the requirement for informed consent was waived.
**Ethical approval:** This study was approved by the Institutional Review Board of Chonnam National University (no. 1040198-210923-HR-146-01).

## 3 Results

Overall, 251,995 patients with disabilities lived and 7,151 died. In terms of severity, 80.2% of disabilities were classified as mild and 19.8% as severe in the non-physical activity group, with a 1.8% higher prevalence of severe disabilities compared to that in the physical activity group. In the non-physically active group, 68.4% were men and 31.6% were women, with an approximately 1.82 times higher prevalence of women compared to that in the physical activity group. The mean ages of the physically inactive and active groups were 64.5 (±13.20) and 58.9 (±12.34) years, respectively. Income levels were three times higher in the non-physically active group than in the physically active group, with a medical aid prevalence of 2.7%. The mean CCI score was 5.7 (±3.24) for the non-physical activity group and 5 (±3.21) for the physical activity group (Table 1).

**Table 1:** Socioeconomic characteristics according to physical activity of persons with disabilities

		Physical activity (150 min per week)				*p*
		<150		≥150		
		*n* (mean)	% (SD)	*n* (mean)	% (SD)	
Severity of disability	Mild	158,866	80.2%	50,052	82.0%	<0.001
	Severe	39,243	19.8%	10,985	18.0%	
Sex	Male	135,500	68.4%	50,465	82.7%	<0.001
	Female	62,609	31.6%	10,572	17.3%	
Age (years)	64.6 ± 13.20	58.9 ± 12.34			<0.001
Health insurance type	Medical aid	5,349	2.7%	561	0.9%	<0.001
	Region (no work)	26,401	13.3%	5,040	8.3%	
	Workplace	166,359	84.0%	55,436	90.8%	
Income quintile	Medical aid	5,349	2.7%	561	0.9%	<0.001
	1	46,483	23.5%	11,228	18.4%	
	2	35,932	18.1%	11,712	19.2%	
	3	33,614	17.0%	10,841	17.8%	
	4	37,199	18.8%	12,717	20.8%	
	5	39,532	20.0%	13,978	22.9%	
Reemployment	No	196,143	99.0%	60,458	99.1%	0.223
	Yes	1,966	1.0%	579	0.9%	
Smoking	No	153,052	77.3%	45,227	74.1%	<0.001
	Yes	45,057	22.7%	15,810	25.9%	
Drinking	No	145,546	73.5%	38,565	63.2%	<0.001
	Yes	52,563	26.5%	22,472	36.8%	
Charlson comorbidity index score		5.7 ± 3.24		5.0 ± 3.21		<0.001
Survival		191,765	76.1%	60,230	23.9%	<0.001
Death		6,344	88.7%	807	11.3%	
Total		198,109	76.4%	61,037	23.6%	<0.001

Note. *n*: number; SD: standard deviation. Adjusting: disability types, region of residence.

The only case in which people with hepatic dysfunction (or liver dysfunction) exercised more than 150 min per week was among the disabled group (51.5%). The frequency of the disability type in participants who exercised for less than 150 min per week was as follows: autistic disorder, 52.7%; intestinal fistula/urinary fistula dysfunction, 55.7%; visual disability, 56.5%; facial disfigurement, 57.1%; speech disability, 57.8%; hearing disability, 58.8%; physical disability, 58.9%; cardiac dysfunction, 59.3%; kidney dysfunction, 61%; epilepsy dysfunction, 63.7%; brain lesion disability, 63.8%; respiratory dysfunction, 65.8%; intellectual disability, 67.5%; and mental disorder, 71.2% (Table 2).

**Table 2:** Analysis of frequency according to disability types and physical activity

		Physical activity (150 min per week)				*p*
		<150		≥150		
Variables		*n*	%	*n*	%	
Disability types	Physical disability	99,241	58.9%	36,119	41.1%	<0.001
	Disability of brain lesion	9,173	63.8%	2,219	36.2%	
	Visual disability	20,768	56.5%	6,804	43.5%	
	Hearing disability	45,456	58.8%	9,947	41.2%	
	Speech disability	1,279	57.8%	408	42.2%	
	Intellectual disorder (mental retardation)	9,121	67.5%	2,224	32.5%	
	Autistic disorder	514	52.7%	269	47.3%	
	Mental disorder	2,686	71.2%	542	28.8%	
	Kidney dysfunction	6,213	61.0%	1,388	39.0%	
	Cardiac dysfunction	350	59.3%	98	40.7%	
	Respiratory dysfunction	861	65.8%	139	34.2%	
	Hepatic dysfunction (or liver dysfunction)	930	48.5%	378	51.5%	
	Facial disfigurement	272	57.1%	132	42.9%	
	Intestinal fistular/urinary fistular dysfunction	877	55.7%	273	44.3%	
	Epilepsy dysfunction	368	63.7%	97	36.3%	
Total		198,109	59.9%	61,037	40.1%	<0.001

Note. *n*: number.

Survival analyses were performed to analyze factors affecting the mortality risk, and the maximum follow-up period was 4 years and 9 months. Those who exercised for more than 150 min per week had a 0.727 times (adjusted hazard ratio [AHR]) lower risk of death than that had by those who exercised less (95% confidence interval [CI]: 0.674–0.784). Females with a disability were 0.726 times (AHR) less likely to die than males (95% CI: 0.685–0.769), and the risk of death was 1.08 times (AHR) higher with increasing age (95% CI: 1.077–1.083). Higher-income quintiles were associated with a lower risk of death, with the 5th quintile having a 0.603-times (AHR) lower risk of death compared to the medical aid group (95% CI: 0.539–0.674). Regarding reemployment, employed patients had a 0.672-times (AHR) lower risk of death than that of unemployed patients (95% CI: 0.457–0.988). Smokers had a 1.487-times (AHR) higher risk of death (95% CI: 1.396–1.583) than that of non-smokers, and increasing CCI scores were linked to a 1.228-times (AHR) higher risk of death (95% CI: 1.22–1.237).

People with visual disabilities had a 1.444-times (AHR; 95% CI: 1.318–1.582) higher risk of death than people with physical disabilities. Likewise, people with hearing disabilities (AHR: 1.792; 95% CI: 1.679–1.913), hepatic dysfunction (AHR: 2.673; 95% CI: 2.115–3.378), speech disabilities (AHR: 2.973; 95% CI: 2.289–3.863), cardiac dysfunction (AHR: 3.003; 95% CI: 2.073–4.351), and brain lesion disabilities (AHR: 3.028; 95% CI: 2.778–3.301) had a higher risk of death. Those with kidney function disability (AHR: 3.457; 95% CI: 3.112–3.841), intellectual disability (AHR: 3.619; 95% CI: 2.899–4.517), intestinal fistula/urinary fistula (AHR: 3.631; 95% CI: 3.043–4.334), mental disability (AHR: 4.22; 95% CI: 3.328–5.351), epilepsy (AHR: 4.453; 95% CI: 2.721–7.29), and respiratory dysfunction (AHR: 5.321; 95% CI: 4.411–6.42) had a higher risk of death (Table 3).

**Table 3:** Survival analysis of mortality risk by physical activity in persons with disabilities

		Univariate model		Multivariate model	
Variables		Crude HR	95% CI	Adjusted HR	95% CI
Physical activity (150 min a week)	<150	1		1	
	≥150	0.416	0.386–0.447	0.727	0.674–0.784
Disability types	Physical disability	1		1	
	Disability of brain lesion	5.205	4.793–5.654	3.028	2.778–3.301
	Visual disability	1.509	1.378–1.653	1.444	1.318–1.582
	Hearing disability	4.392	4.143–4.656	1.792	1.679–1.913
	Speech disability	2.189	1.686–2.841	2.973	2.289–3.863
	Intellectual disorder (mental retardation)	0.532	0.432–0.656	3.619	2.899–4.517
	Autistic disorder	0	0–2.59	0.001	0–2.358
	Mental disorder	1.806	1.439–2.266	4.22	3.328–5.351
	Kidney dysfunction	6.173	5.636–6.761	3.457	3.112–3.841
	Cardiac dysfunction	3.848	2.667–5.551	3.003	2.073–4.351
	Respiratory dysfunction	7.528	6.295–9.003	5.321	4.411–6.42
	Hepatic dysfunction (or liver dysfunction)	3.444	2.731–4.343	2.673	2.115–3.378
	Facial disfigurement	0.575	0.216–1.533	1.517	0.568–4.047
	Intestinal fistula/urinary fistula dysfunction	8.604	7.225–10.246	3.631	3.043–4.334
	Epilepsy dysfunction	1.919	1.173–3.138	4.453	2.721–7.29
Severity of disability	Mild	1		1	
	Severe	0.945	0.894–1	0.856	0.799–0.917
Sex	Male	1		1	
	Female	1.056	1.001–1.113	0.726	0.685–0.769
Age	Per year	1.098	1.096–1.101	1.08	1.077–1.083
Income quintile	Medical aid	1		1	
	1	0.238	0.213–0.266	0.509	0.454–0.57
	2	0.219	0.195–0.245	0.499	0.444–0.562
	3	0.239	0.213–0.268	0.633	0.562–0.712
	4	0.233	0.208–0.261	0.62	0.552–0.697
	5	0.384	0.345–0.428	0.603	0.539–0.674
Reemployment	No	1		1	
	Yes	0.491	0.334–0.722	0.672	0.457–0.988
Smoking	No	1		1	
	Yes	0.726	0.685–0.769	1.487	1.396–1.583
Drinking	No	1		1	
	Yes	0.449	0.422–0.478	0.945	0.883–1.012
CCI score	M (SD)	1.347	1.339–1.354	1.228	1.22–1.237

Note. CCI: Charlson comorbidity index; CI: confidence interval; HR: hazard ratio; M: mean; SD: standard deviation. Adjusting: region of residence.

## 4 Discussion

In this study, only 40.1% of the PWDs met the WHO guidelines of more than 150 min of physical activity per week. The 2020 revised WHO guidelines set the recommended level of physical activity for PWDs at 150 min or more per week, which was the same for persons without disabilities [5]. This study also found that among PWDs, those with higher levels of physical activity had lower mortality rates (crude hazard ratio [CHR]: 0.416, 95% CI: 0.674–0.784). Therefore, the high mortality rates of those with disabilities could be partly related to low physical activity. The relationship between physical activity levels and death was also significant when adjusting for other factors related to physical activity levels, such as disability severity, disability type, income level, smoking, and alcohol consumption (AHR: 0.727, 95% CI: 0.674–0.784). These results therefore indicate that policies may be needed to increase physical activity levels and consequently lower mortality among PWDs. Regular physical activity can help improve health, maintain physical function, and improve the quality of life with little time and cost [16].

Meanwhile, a study on exercise participation among PWDs found that only 40% of PWDs exercise regularly [2]. Most participants who do not exercise regularly are severely disabled and are highly constrained by the lack of desired programs and a fear of injury during exercise. Therefore, in the future, customized exercise programs for PWDs should be developed to ensure safety by considering the risks that may arise from various disability characteristics. In addition, compensation for exercise differences by income is lacking [17], and this needs to be complemented by policy.

This study only included PWDs who participated in health screenings and reported their physical activity. Therefore, PWDs who participated in health screenings and indicated their physical activity levels are assumed to have relatively high levels of health [18]. Caution should be exercised when considering groups of PWDs who do not routinely receive health checks or indicate their physical activity levels, as lower actual physical activity levels may have a greater impact on mortality.

Another notable finding is the relationship between other health risk behaviors and mortality. In another study, smoking contributed to less physical activity in PWDs [19]. In this survival analysis, when only smoking was considered, smoking was not significantly associated with mortality (CHR: 0.726, 95% CI: 0.685–0.769), but when other factors were adjusted, smoking increased the risk of death (AHR: 1.487, 95% CI: 1.396–1.583). Introducing policies that simultaneously change health behaviors, such as combined interventions that improve smoking and physical activity, could further reduce mortality.

PWDs have poorer health behaviors than people without disabilities owing to their circumstances [9]. PWDs have difficulty participating in exercise and physical activities because of issues with accessibility, economic problems, physical problems, professional leadership problems, and lack of laws and institutional devices [16]. PWDs are more likely to be physically inactive and obese and have diabetes, metabolic syndrome, and lipid disorders, all of which are risk factors for cardiovascular diseases [10]. High levels of physical activity are associated with various health benefits. Physical activity prevents cardiovascular diseases, such as heart disease, high blood pressure, and cerebrovascular disease, and chronic diseases, such as diabetes and cancer, by enhancing cardiopulmonary function and muscle strength [18]. Furthermore, regular participation in physical activity can help maintain independence and prevent secondary health problems caused by disability [20]. These findings emphasize the need to develop and implement policies to increase physical activity levels among PWDs by making the appropriate environments.

In addition, the ability of PWDs to be physically active may vary depending on the type and severity of their disability. For example, the types of physical activity performed by the groups with respiratory and visual impairments differ, and the ability to perform physical activity vary, even in those with severe and mild disabilities [21]. Therefore, to ensure an appropriate level of physical activity among those with disabilities, developing a program tailored to the type and degree of disability is necessary.

A limitation of this study was that owing to limited data, the role of sedentary behavior, which is expected to have increased during the pandemic, was not included in the physical activity questionnaire. Furthermore, although the WHO has defined sedentary behavior, whether moderate-intensity activity is included is unclear. Future studies should include these variables to measure physical activity.

## 5 Conclusion

This study using data representative of the population of South Korea demonstrated that the low level of physical activity among the group with disabilities is closely related to high mortality levels, after adjusting for other factors that may be related to physical activity and mortality. Males; older adults; PWDs such as respiratory, epilepsy, and mental disorders; people with a low income; unemployed patients; smokers, and patients with high CCI scores are at higher risk of death. Our study confirmed that physical inactivity affects mortality after adjusting for CCI scores, smoking, and alcohol consumption among PWDs. Thus, it is important to increase physical activity to prevent complications and increase longevity in PWDs. Therefore, it is necessary to develop customized exercise support policies that consider disability-specific characteristics and severity for populations at high risk of mortality.

## Abbreviations

AHR: adjusted hazard ratio
CCI: Charlson comorbidity index
CI: confidence interval
NHIS: National Health Insurance Service
PWDs: persons with disabilities
WHO: World Health Organization
